# Ecological and Morphological Profile of Floating Spherical *Cladophora socialis* Aggregations in Central Thailand

**DOI:** 10.1371/journal.pone.0124997

**Published:** 2015-04-21

**Authors:** Isao Tsutsui, Tatsuo Miyoshi, Halethichanok Sukchai, Piyarat Pinphoo, Dusit Aue-umneoy, Chonlada Meeanan, Jaruwan Songphatkaew, Sirimas Klomkling, Iori Yamaguchi, Monthon Ganmanee, Hiroyuki Sudo, Kaoru Hamano

**Affiliations:** 1 Fisheries Division, Japan International Research Center for Agricultural Sciences (JIRCAS), Tsukuba, Ibaraki, Japan; 2 Research Center for Marine Invertebrates, National Research Institute of Fisheries and Environment of Inland Sea, Onomichi, Hiroshima, Japan; 3 Shrimp Co-culture Research Laboratory (SCORL), King Mongkut’s Institute of Technology Ladkrabang (KMITL), Bangkok, Thailand; 4 Department of Animal Production and Fisheries, King Mongkut’s Institute of Technology Ladkrabang (KMITL), Bangkok, Thailand; 5 Department of Life, Environment and Materials Science, Fukuoka Institute of Technology (FIT), Fukuoka, Japan; National Taiwan Ocean University, TAIWAN

## Abstract

The unique beauty of spherical aggregation forming algae has attracted much attention from both the scientific and lay communities. Several aegagropilous seaweeds have been identified to date, including the plants of genus *Cladophora* and *Chaetomorpha*. However, this phenomenon remains poorly understood. In July 2013, a mass occurrence of spherical *Cladophora* aggregations was observed in a salt field reservoir in Central Thailand. The aims of the present study were to describe the habitat of the spherical aggregations and confirm the species. We performed a field survey, internal and external morphological observations, pyrenoid ultrastructure observations, and molecular sequence analysis. Floating spherical *Cladophora* aggregations (1–8 cm in diameter) were observed in an area ~560 m^2^, on the downwind side of the reservoir where there was water movement. Individual filaments in the aggregations were entangled in each other; consequently, branches growing in different directions were observed within a clump. We suggest that water movement and morphological characteristics promote the formation of spherical aggregations in this species. The molecular sequencing results revealed that the study species was highly homologous to both *C*. *socialis* and *C*. *coelothrix*. However, the diameter of the apical cells in the study species was less than that of *C*. *coelothrix*. The pyrenoid ultrastructure was more consistent with that of *C*. *socialis*. We conclude that the study species is *C*. *socialis*. This first record of spherical aggregations in this species advances our understanding of these formations. However, further detailed physical measurements are required to fully elucidate the mechanism behind these spherical formations.

## Introduction

The unique morphology of spherical growth seaweeds has gained them considerable popularity [1], both socially and scientifically. Mass media have sensationally referred to sporadic occurrences of aegagropilous seaweeds as mystery balls, UFOs, and green alien balls [2, 3]. An extremely rare freshwater alga, *Aegagropila linnaei*, is known for its beautiful spherical filamentous aggregations [4]. It has become popular in the souvenir and aquarium trades, and is depicted on postage stamps from Japan and Iceland [1, 5]. This alga is an endangered or protected species in Japan, Iceland, United Kingdom, Germany, Sweden, and Russia, because of declining populations, and is included in national red lists and other conservation strategies [5, 6]. Scientifically, however, the mechanism for forming spherical aggregations has been researched and discussed. It is generally assumed that the ball shape is a result of both mechanical factors, such as wave-induced rolling motion, and morphological features leading to entanglement of the filament [1, 5].

Besides *A*. *linnaei*, several aegagropilous seaweeds are known [7–9], e.g., at least 18 green, 11 brown, and 25 red algae form aggregations [2]. Of the Cladophorales family species, *Chaetomorpha picquotiana* [2], *Cladophora coelothrix* [10], *C*. *prolifera* [9, 11, 12] have ball-like forms including loose-lying balls and spherical forms. However, information on the habitat and ecology of spherical aggregation forms of this taxon remains rare.

*Cladophora* is one of the largest and most common green algal genera, having a worldwide distribution [13]. Despite having simple external morphologies, there are 242 recognized species in this genus [14]. However, it is difficult to identify the species in this genus by external morphology, because of its few morphological characteristics and extensive morphological plasticity related to environmental conditions, age of the alga, and season [15–18]. Additionally, *Cladophora* is not monophyletic, and even the same morphospecies are distributed in a number of different clades in a phylogenetic tree, as a result species identification using only nucleic acid sequences is very difficult [13, 19, 20]. Thus, various methods are required to identify the *Cladophora* species [21, 22], including internal morphology and ultrastructure [18, 22–25], external morphology, and molecular sequencing analysis.

The aims of this study were: 1) to describe the habitat of the spherical *Cladophora* aggregations that occurred in July 2013 in Central Thailand and 2) to use a combination of external and internal morphological characteristics, ultrastructure, growth pattern, and molecular sequence analysis to identify the aggregations to the species level.

## Materials and Methods

### Ethics Statement

All research activities in this study, including field surveys, observations, and laboratory experiments, were permitted by the National Research Council of Thailand (NRCT) (Project ID 2011/005). The field survey site was a privately owned pond and the authors obtained oral approval for sampling from the pond owner. The field study did not involve endangered or protected species.

### Field survey

**Fig 1:**
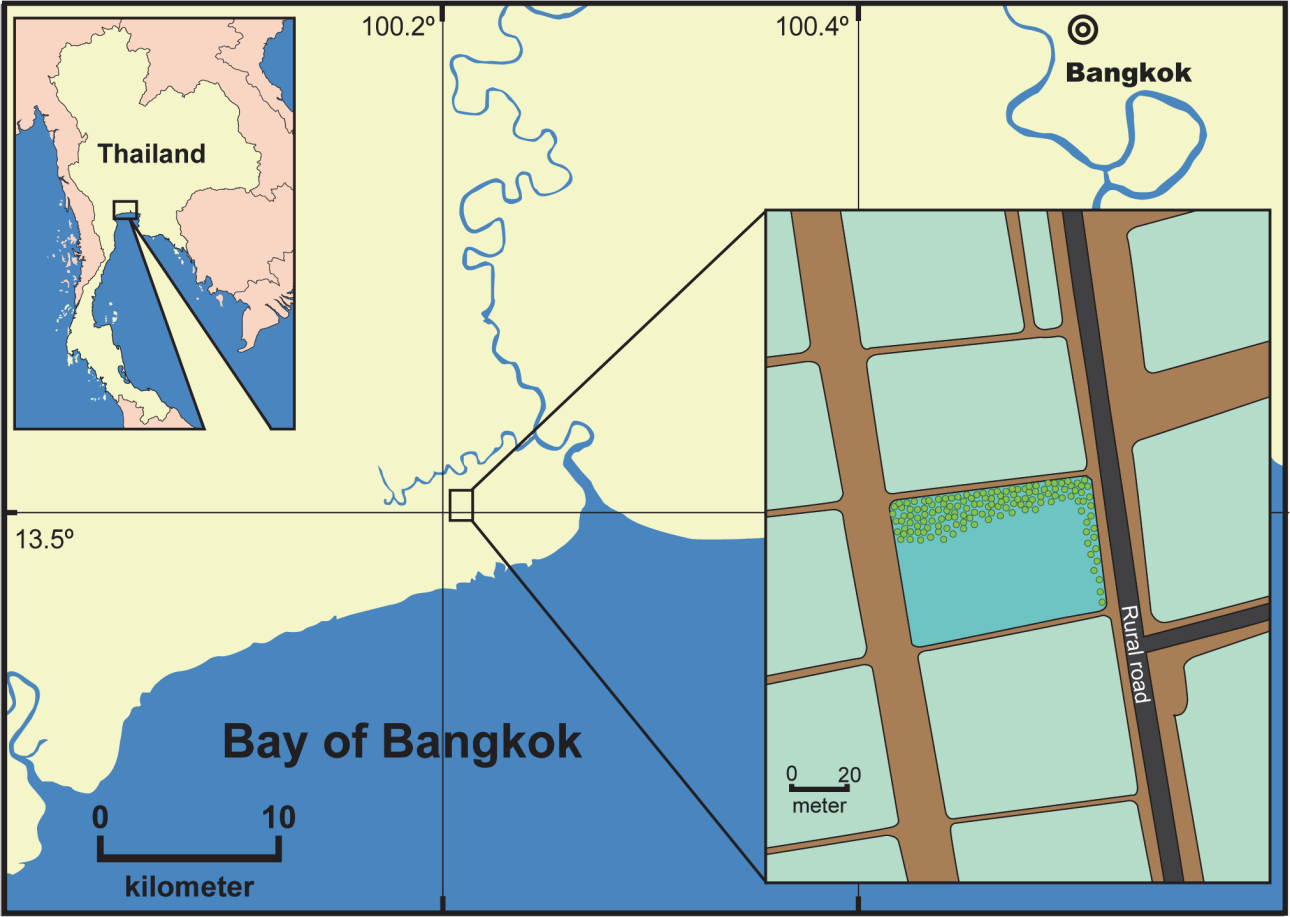
Map showing field survey location in Central Thailand.

A field survey was conducted in a salt field reservoir in Samut Sakhon Province (13.504629N and 100.217659E) to identify the habitat of spherical *Cladophora* aggregations in July 2013 during a large-scale occurrence (Fig 1). Salinity and water temperature were measured on site with a conductivity meter (Condo 3210, WTW Inc., Weilheim, Germany). The pond area was measured with a laser distance meter (GLM 250 VF, Robert Bosch Inc., Stuttgart, Germany). A plastic ruler was used to measure water depth. Total coverage of floating spherical aggregations was directly observed by eye and estimated. The water movement was analyzed from video images of seaweed movement captured by mobile phone (GT-i9500, Samsung Electronics Co., Ltd., Suwon, South Korea). A small number of spherical aggregations of this alga were collected by hand at the survey site and immediately placed in plastic bags with water, after which they were transported to the Shrimp Co-culture Research Laboratory (SCORL), Faculty of Agricultural Technology, King Mongkut’s Institute of Technology Ladkrabang (KMITL), Thailand for observation and laboratory experiments. Some of the samples were dried, pressed, and preserved in the herbarium as specimens at SCORL.

### Morphological observations

Samples brought back to SCORL were rinsed in artificial seawater (Rhoto Marine II, Rei-Sea Co. Ltd., Tokyo, Japan) with a salinity of 20 PSU. Part of the spherical aggregations was cut off and observed under an optical microscope (BX 41, Olympus Co. Ltd., Tokyo, Japan) to study the external morphology. For internal morphology, such as nuclei, the samples were fixed with 3:1 ethanol: glacial acetic acid and stained with aceto-iron-hematoxylin chloral hydrate [26].

### Ultrastructure observations

The samples were fixed with 2% paraformaldehyde (PHA) and 2% glutaraldehyde (GA) in 0.05 M cacodylate buffer pH 7.4 at 4°C overnight. After fixation, the samples were washed three times with 0.05 M cacodylate buffer for 30 min, and postfixed with 2% osmium tetroxide (OsO_4_) in 0.05 M cacodylate buffer at 4°C for 3 h. The samples were dehydrated in graded ethanol solutions; 50% for 30 min at 4°C, 70% for 30 min at 4°C, 90% for 30 min at room temperature, 100% ethanol was changed four times at room temperature. After the dehydration process, the samples were continuously dehydrated in 100% ethanol at room temperature overnight. The samples were infiltrated with propylene oxide (PO) twice for 30 min and put into a 7:3 mixture of PO and resin (Quetol-651, Nisshin EM Co., LTD., Tokyo, Japan) for 1 h. The tube cap was left open overnight and PO was volatilized. The samples were transferred to fresh 100% resin and polymerized at 60°C for 48 h. The polymerized resins were ultra-thin sectioned at 70 nm with a diamond knife using an ultra-microtome (Ultracut UCT, Leica, Vienna, Austria), and the sections were mounted on copper grids. These samples were stained with 2% uranyl acetate at room temperature for 15 min, and washed with distilled water followed by secondary staining with lead stain solution (Sigma Aldrich Co. Ltd., Tokyo, Japan) at room temperature for 3 min. The samples on grids were observed on a transmission electron microscope (JEM-1400Plus, JEOL ltd., Tokyo, Japan) at an acceleration voltage of 80 kV. Digital images were taken with a CCD camera (VELETA, Olympus Soft Imaging Solution GMBH, Münster, Germany).

### Growth pattern experiment

Prior to the experiments, powdered, artificial seawater (Rhoto Marine II, Rei-Sea Co. Ltd., Tokyo, Japan) was diluted with distilled water and adjusted to 20 PSU with a conductivity meter (Condo 3210, WTW Inc.). This experimental medium was sterilized at 121°C for 20 min in an autoclave (HVE-50, Hirayama Manufacturing Corp., Saitama, Japan), after which the pH was adjusted to 8.0 with NaOH using a pH meter (SevenGo, Mettler Toledo, Schwerzenbach, Switzerland). As a nutrient source, a ratio of 3 mL of stock solution of Provasoli’s enriched seawater (PES) in 1 L artificial seawater (approximately 1/6 concentration of PES) was used [27], germanium dioxide (3 mg L^−1^) was added to prevent diatom growth [28]. A small clump of seaweed was kept in a 50 mL conical tube with distilled artificial seawater and oscillated to separate it into small particles with a mixer (VTX-3000L, LMS Co, Ltd, Tokyo, Japan) for 1 min. Healthy filaments without epiphytes or damage, measuring 1.5 mm in length were selected with a stereomicroscope (SZX12, Olympus Co. Ltd.) for the growth pattern experiment. Filaments were individually placed in a 6-well cell culture plate (approximately 4 cm in diameter per well) with 10 mL of medium. The plate was kept at room temperature (approximately 29–30°C) with a 12:12 h light: dark cycle and a photon flux density of 340 μmol m^−2^ s^−1^. Algal development was observed every morning under the stereomicroscope for 7 days.

### Molecular sequencing

Samples were frozen in liquid nitrogen until use and DNA was extracted with a DNeasy Plant Mini Kit (Qiagen Sciences, Germantown, MD, USA) according to the manufacturer’s instructions. The isolated DNA was used for PCR amplification of ribosomal RNA genes using the 18S rDNA specific primers SR-1f (5′-TACCTGGTTGATCCTGCCAG-3′) and 18S-C2r (5′-TCCGCAGGTTCACCTACGGAG-3′) [29] and the 28S rDNA specific primers C1FL (5′-ACCCGCTGAACTTAAGCATATC-3′) and D2FL (5′-GGTCCGTGTTTCAAGACGG-3′) [20]. The PCR products were purified with a NucleoSpin Gel and PCR Clean-up Kit (Macherey-Nagel Inc., Düren, Germany) and subcloned into a pCR4-TOPO vector using a TOPO TA Cloning Kit for sequencing (Thermo Fisher Scientific Inc., Weltham, MA, USA). Sequencing was carried out by Eurofins Genomics K.K., Tokyo, Japan. The 18S rDNA and 28S rDNA sequences of this alga were submitted to the DNA Data Bank of Japan (DDBJ) and were searched for homology using the program BLAST at the DDBJ. This nucleotide sequence and the published sequences of other filamentous algae were aligned by Clustal W [29]. The Maximum likelihood (ML) and maximum parsimony (MP) algorithms were used to construct a phylogenetic tree. Bootstrap values were obtained from 500 resamplings. *Cladophora herpestica* (Z35419) (syn. *Cladophoropsis zollingerii*) and *Cladophora sericea* (Z35320) were used as out-groups for the 18S rDNA phylogenetic tree, and *C*. *herpestica* (AM503460) and *C*. *sericea* (AM503474) were used as out-groups for the 28S rDNA phylogenetic tree, based on Leliaert et al. [20].

## Results

### Chronology

A mass occurrence of spherical *Cladophora* aggregations was observed in a salt field reservoir in Samut Sakhon Province in July 2013 (Fig 1). Thereafter, only a few residual spherical aggregations were observed and all had disappeared by August. Spherical aggregation blooms do not occur every year, and have only been observed twice (2010 and 2013) in at least 50 years in this area, according to the pond owner (pers. comm. Mr. and Mrs. Yasathon).

### Habitat

**Fig 2:**
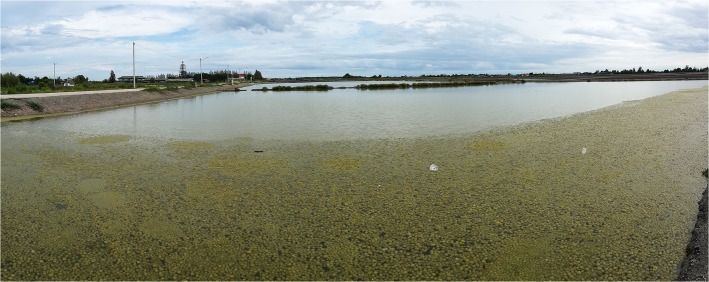
Mass floating spherical aggregations of *Cladophora socialis* in a salt field reservoir in Central Thailand.

**Fig 3:**
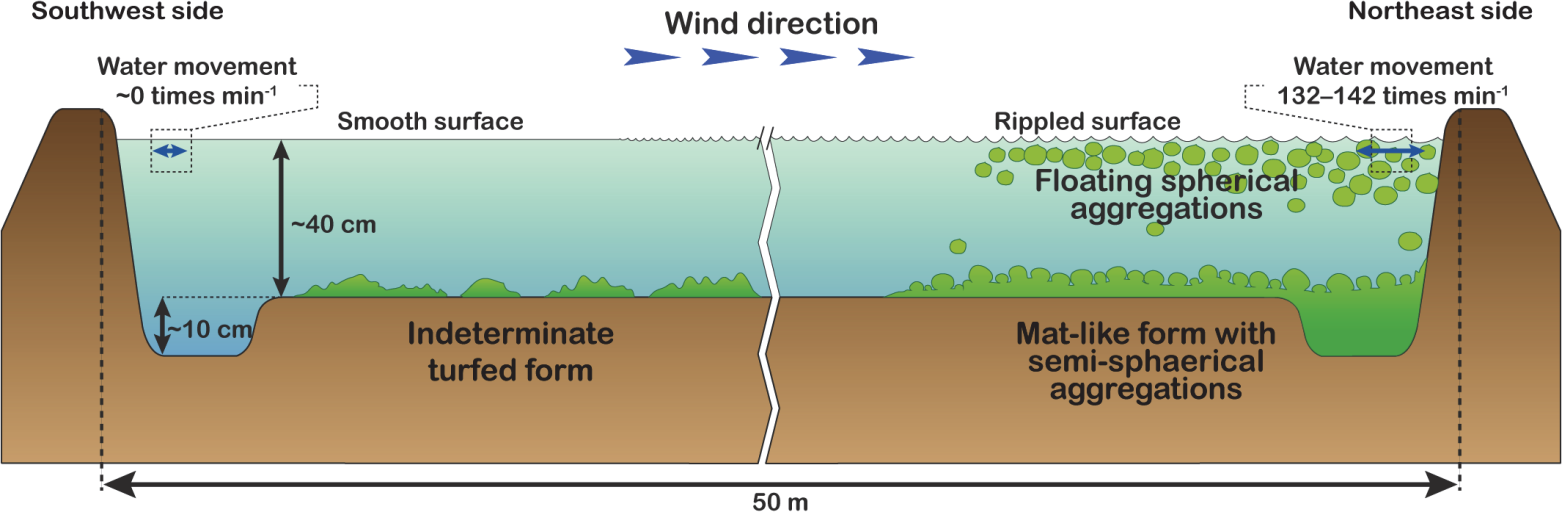
Transverse schematic diagram showing the location of several *Cladophora socialis* forms at the study site.

**Fig 4:**
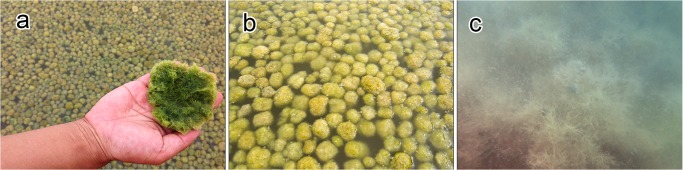
Floating spherical aggregations of *Cladophora socialis* in a salt field reservoir, a natural habitat. (a) A lower hemisphere of spherical aggregations of a solid, dark green color: (b) On-land view of floating spherical aggregations: (c) Underwater view of Indeterminate turf forms.

**Fig 5:**
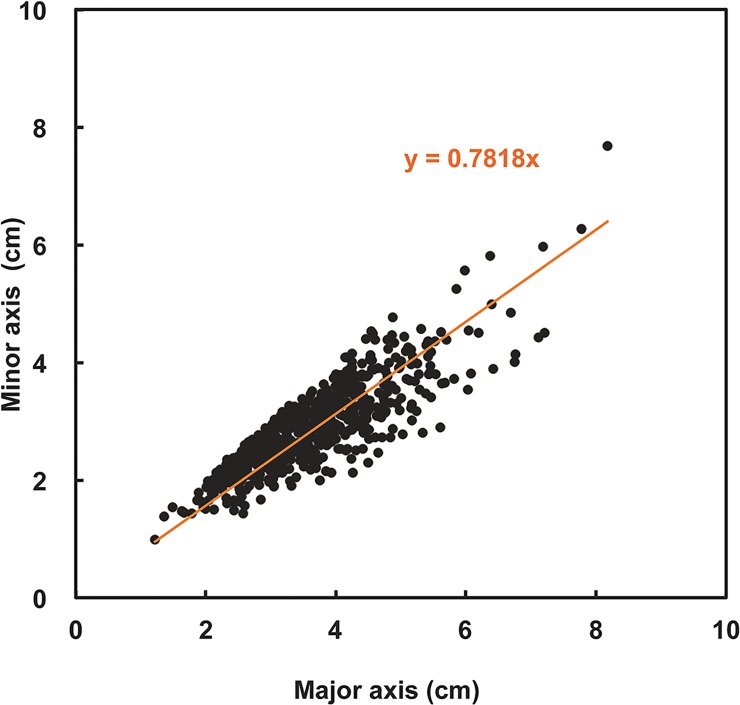
Scattergraph showing the size of spherical *Cladophora socialis* (major and minor axis) aggregations.

The reservoir where the mass of spherical aggregations occurred was approximately 70 m long, 50 m wide, and 40–50 cm deep. Water temperature, salinity, and pH at the reservoir were 32.8°C, 35 PSU, and 8.97, respectively. Extensive populations of spherical aggregations floated, accumulated, and colonized on the water surface close to the shore downwind in an area of approximately 560 m^2^ with a density of 706 aggregations m^−2^ (Fig 2). Aggregations were slightly rolling and trembling horizontally 132–142 times per min by wind-generated ripples (Table 1 and Fig 3 and S1 Movie). Individual aggregations were soft, solid, with irregularly entangled filaments, and dark green in color (Fig 4A and 4B). They ranged in diameter from 1.2–8.2 cm (mean 3.6 cm) along the major axis and 1.0–7.7 cm (mean 2.9 cm) along the minor axis (Fig 5). Mat-like forms with semi-spherical aggregations (not yet completely aggregated) were observed attached to or lying on the clay bottom offshore on the downwind side of the reservoir (S1 Movie). However, indeterminate turfed forms were observed loosely attached to or lying on the clay bottom offshore at the windward side of the reservoir, where there was almost no water movement (Fig 4C).

**Table 1:** Outline of habitat and habit of several Cladophora socialis forms at the study site.

	Southwest side	Northeast side
Aggregation form	Indeterminate turfed form	Floating spherical aggregation, mat-like forms with semi-spherical aggregation
Water surface	Calm and smooth	Ripple with wind
Water movement (times m^−1^)	~0	132–142
Position related to wind direction	Windward	Downwind
Water depth (cm)	40–50	40–50

### External and internal morphology

**Fig 6:**
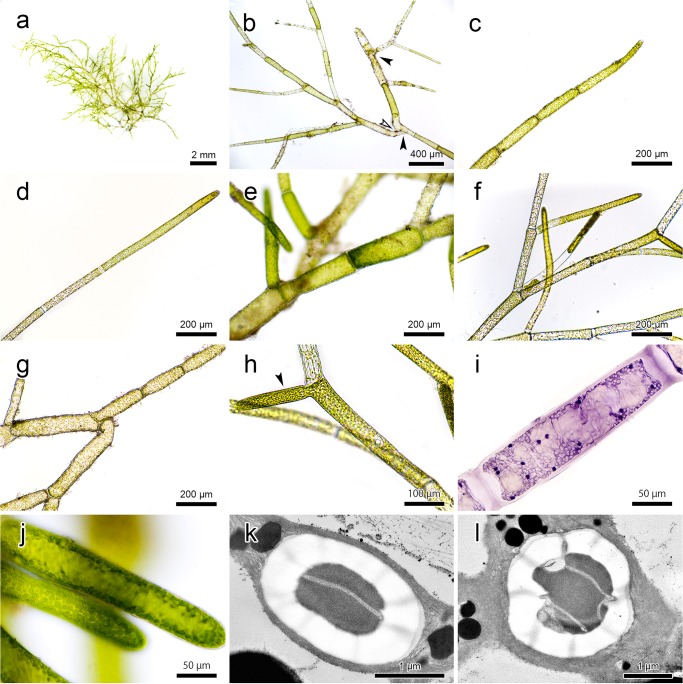
Morphological characteristics of *Cladophora socialis*. (a) A fresh individual thallus: (b) Filaments attaching to one another by rhizoids formed apically (filled-arrow) and intercalary (open-arrow): (c) Apical cell in natural individual: (d) Apical cell cultured in static water showing longer length: (e) Main axis cells: (f) Branching system: (g) Filaments showing different growing directions because of a bent obtuse angle: (h) Newly formed lateral (filled-arrow) without cross-wall at its base: (i) Multi-nuclei in a cell stained by the Wittmann method: (j) Parietal chloroplasts along the cell wall: (k) Transmission electron microscopic image of bilenticular type pyrenoid: (l) Transmission electron microscopic image of zonal type pyrenoid.

Individual filaments were green to light green and measured 2–3 cm in total length when fully developed (Fig 6A). Rhizoids were formed apically or intercalary and attached to another filament (Fig 6B); consequently, branches within a clump grew in different directions. The apical cells were cylindrical, round ended, 27.7–42.6 μm (mean = 36.0, SD ± 4.84) in diameter, 7.2–16.5 (9.44 ± 1.92) in length/diameter (L/D) ratio, and 0.9–2.6 μm (1.5 ± 0.41) in cell wall thickness (Fig 6C and 6D). The main axis cells were straight or slightly curved, cylindrical, 60.2–99.6 μm (72 ± 6.54) in diameter, 3.3–7.1 (4.8 ± 0.96) in length/diameter (L/D) ratio, and 1.7–4.6 μm (2.7 ± 0.62) in cell wall thickness (Fig 6E). Branching angles were normally 23.8–70.6° (47.7° ± 11.82), and were occasionally close to 180°, with single and sometimes double branching systems (Fig 6F and 6G). Newly formed laterals inclined to without cross-walls at their base (Fig 6H). The cells were multi-nucleate, with 12–38 (23.3 ± 6.65) nuclei in apical cells and 10–66 (23.5 ± 11.08) in main axis cells (Fig 6I). The nuclei major axes were 3.2–5.7 μm (4.2 ± 0.59) and 2.1–7.2 μm (3.6 ± 1.02) in apical and main axis cells, respectively. The chloroplasts were parietal along the cell wall (Fig 6J).

### Pyrenoid ultrastructure

The pyrenoids were spherical to ellipsoidal: 1.2–3.1 μm (2.3 ± 0.45) along the major axis, 0.8–3.1 μm (1.9 ± 0.48) along the minor, and 1.0–2.4 (1.3 ± 0.24) in L/D ratio. Two different types of pyrenoids were observed; approximately 80% were bilenticular, which was composed of a matrix traversed by a single thylakoid band surrounded by two cup-shaped starch sheaths (Fig 6K), and approximately 20% were zonal, in which the matrix was divided by two or more parallel thylakoid bands and surrounded by three or more starch sheaths (Fig 6L).

### Growth pattern

**Fig 7:**
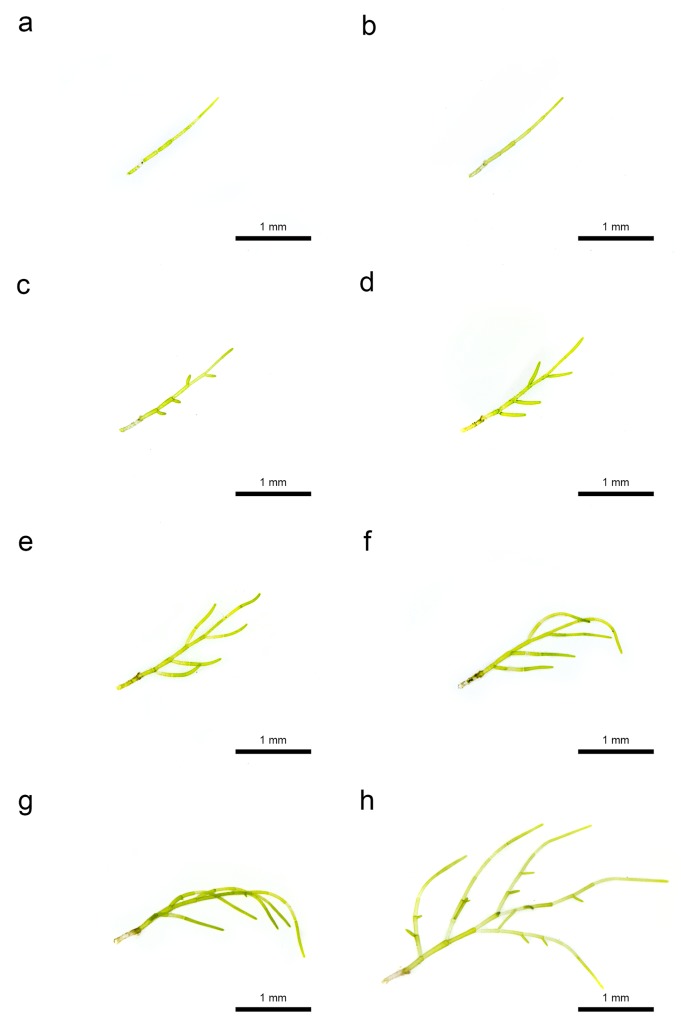
Growth of *Cladophora socialis* in laboratory experiments. (a) Day 0: (b) Day 1: (c) Day 2: (d) Day 3: (e) Day 4: (f) Day 5: (g) Day 6: (h) Day 7.

Filamentary particles, 1.5 mm long, without branching (six cells) were cultured (Fig 7A). Lateral formations were observed at the apical pole of each of the four intermediate cells on the 2^nd^ day (Fig 7C). The cell walls were formed at the basal portion of laterals, and the apical cell divided in two on the 3^rd^ day (Fig 7D). The lateral cells extended, divided, and became branchlets on the 4^th^ and 5^th^ days (Fig 7E and 7F). Lateral formations were observed from branchlets on the 6^th^ day (Fig 7G). The cell walls were formed at the basal portion of new laterals on the 7^th^ day (Fig 7H). Thus, growth of this seaweed was by division of apical and lateral cells, which were produced at intermediate cells.

### Molecular sequencing analysis

**Fig 8:**
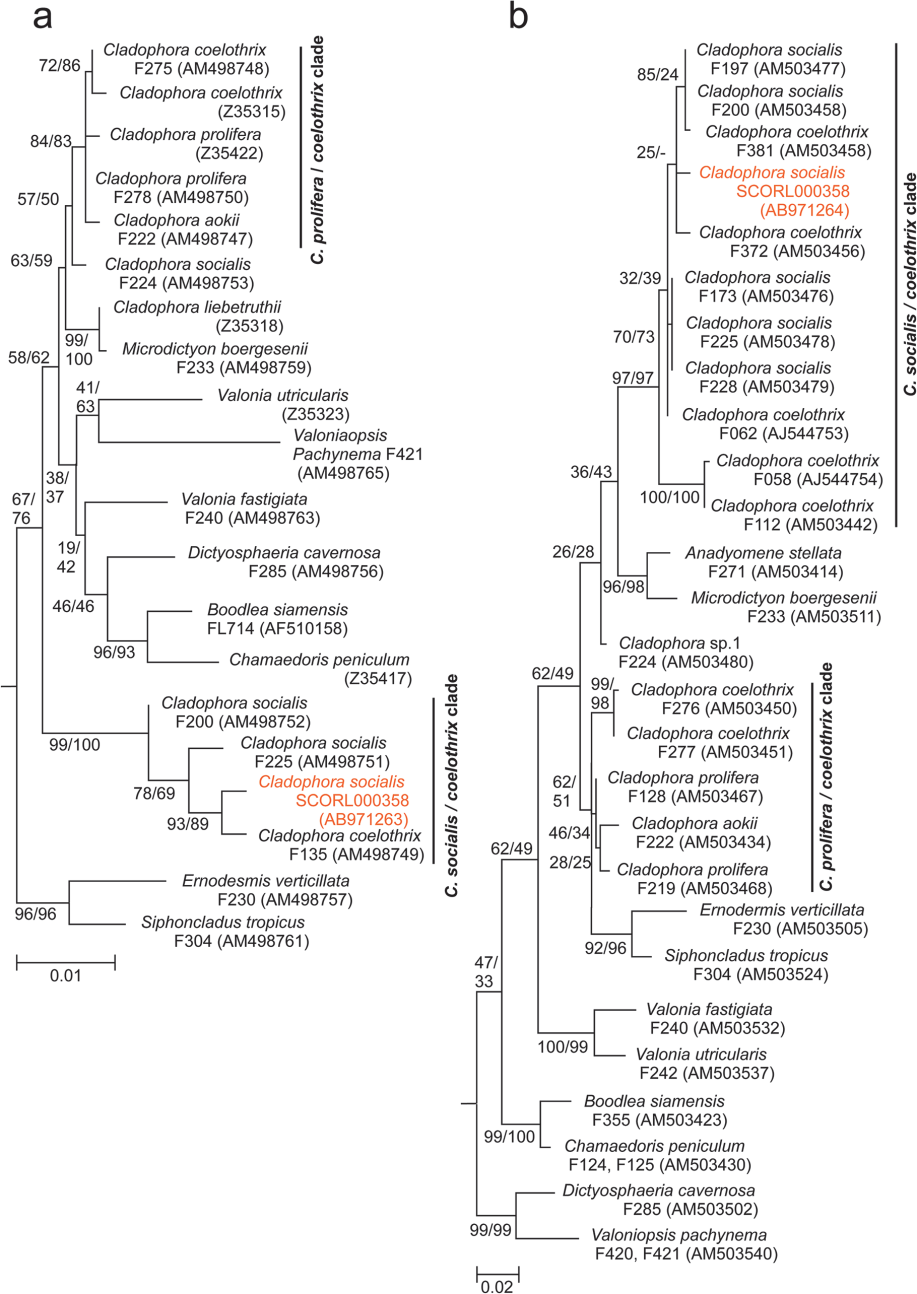
Maximum likelihood phylogenetic trees for *Cladophora socialis* and relatives. (a) A tree based on nearly complete 18S rDNA sequences: (b) A tree based on partial 28S rDNA sequences. Other data on Cladophorales were downloaded from GenBank for comparison. Numbers close to the nodes are ML/MP bootstrap values.

The almost complete 18S rDNA sequence of this alga consisted of 1769 nucleotides. It was registered on DDBJ under the accession number AB971263. Homologies of 18S rDNA between this seaweed and other related seaweed sequences in the DDBJ were 99.5% in *C*. *coelothrix* from Philippines and 99.1% in *C*. *socialis* from Australia and Panama (Table 2 and Fig 8). The partial 28S rDNA sequence of this alga consisted of 608 nucleotides. It was registered on DDBJ under the accession number AB971264. Homologies of 28S rDNA between this seaweed and other related seaweed sequences in the DDBJ were 98.8% in *C*. *socialis* from South Africa, Jamaica, Panama, and Australia (Table 3 and Fig 8).

**Table 2:** Homology of *Cladophora socialis* 18S rDNA sequence to related species.

Species	Voucher	Accession No.	Identities (bp)	Similarity (%)	Locality	Reference
*C*. *socialis*	SCORL 000358	AB 971263	1769		Samut Sakhon, Thailand	Present study
*C*. *coelothrix*	F135	AM498749	1679/1687	99.5	Mactan Is., Philippines	[20]
*C*. *socialis*	F225	AM498751	1673/1688	99.1	Rottnest Is., Australia	[20]
*C*. *socialis*	F220	AM498752	1671/1687	99.1	Balboa, Panama	[20]
*C*. *socialis*	F224	AM498753	1657/1688	98.2	Virgin Is., USA	[20]
*C*. *liebetruthii*		Z35318	1728/1770	97.6	Republic of Cabo Verde	[17]

**Table 3:** Homology of *Cladophora socialis* 28S rDNA sequence to related species.

Species	Voucher	Accession No.	Identities (bp)	Similarity (%)	Locality	Reference
*C*. *socialis*	SCORL 000358	AB971264	608		Samut Sakhon, Thailand	Present study
*C*. *socialis*	F197	AM503477	561/568	98.8	KwaZulu-Natal, South Africa	[20]
*C*. *socialis*	F173	AM503476	561/568	98.8	Portland, Jamaica	[20]
*C*. *socialis*	F200	AM503440	561/581	98.8	Balboa, Panama	[20]
*C*. *socialis*	F225	AM503478	560/567	98.8	Rottnest Is., Australia	[20]
C. *socialis*	F228	AM503479	559/566	98.8	unknown	[20]

## Discussion

Recent molecular phylogenetic studies have proven that *Cladophora* is not a monophyletic genus and that most of the morphological distinctions are untenable [13, 17, 19, 30]. Based on our phylogenetic analysis for both18S rDNA and 28S rDNA, the present species belongs to the Siphonocladales lineage and not Cladophorales. These results support the assertion that some members of the order Cladophorales should be reassigned to new genera and families [13].

It was impossible to determine if the study species is *Cladophora coelothrix* or *C*. *socialis* from our homology results, because the most closely related species were different in respective parts of the nucleic acid sequence, i.e., the study species was highly homologous to *C*. *coelothrix* and *C*. *socialis* in 18S rDNA and to *C*. *socialis* in 28S rDNA. *C*. *socialis* and *C*. *coelothrix* also closely resemble each other in external morphology; however, *C*. *socialis* can be distinguished from *C*. *coelothrix* by its smaller cell diameter, which is approximately half as thick [31–34]. Although the diameter range of the main axes overlapped slightly, that of the apical cells of the study species was much thinner than that of *C*. *coelothrix* (Table 4). Additionally, pyrenoid ultrastructure and growth pattern in the study species was consistent with that of *C*. *socialis* (Table 4). Thus, based on its external morphology, ultrastructure, growth pattern, and the molecular sequencing analysis, we conclude that the study species is *C*. *socialis*.

**Table 4:** Outline of the major morphological characteristics and habitats of *Cladophora socialis* and related *Cladophora* species.

	*Cladophora socialis*	*C*. *coelothrix*			*C*. *socialis*			
**External morphology**								
** Apical cells**								
** **Diameter (μm)	28–43	55–140	57–75	65–120	24–54	30–40	25–50	35–55
** **L/D	7–17	5–16	5–15	2.5–16	7–18	3–5	10–40	4–22
** **Shape of terminal portion	Round		Round	Round			Round	Round
** **Cell wall thickness (μm)	0.9–2.6		1–3	0.5	< 1		1	0.5–1
** Main axis cells**								
** **Diameter (μm)	60–99	60–220	75–120	65–170	44–80	40–60 (70)	35–55	35–85
** **Ratio of Length to Diameter	3–7		2.5–12	1.5–12	2–20	4–5	3–20	3–15
** **Cell wall thickness (μm)	1.7–4.6		< 15	< 4	2–4		< 8	< 3
** Ramification**								
** **Angle	24–70 (180)	< 90	20–50	> 45	45–90		45–90	> 45
**Internal morphology**								
** Chloroplast**								
** **Position	Peripheral reticulate							
** **Morphological form	Plate							
** Nuclei**								
** **No. in apical cells	12–38							
** **No. in main axis cells	10–66							
** **Diameter in apical cells (μm)	3–6							
** **Diameter in main axis cells (μm)	2–7							
**Ultrastructure**								
** Pyrenoid**								
** **Diameter (μm)	1–3							
** **Shape	Spherical, Ellipsoidal							
** **Type	Bilenticular, Zonal							Bilenticular
**Growth pattern**	ACD at terminal cells and lateral division at intermediate cells	ACD	Mainly ACD, ICD lower down	Mainly ACD, ICD in shorter cells	Mainly ACD, ICD in shorter cells	ICD	ICD	Mainly ACD, ICD
**Ecology**								
** **Habitat	Stagnant water	Wave-exposed to mangrove areas	Intertidal to subtidal area	Shady places at sublittoral	Wave-exposed to mangrove areas	Intertidal, tidal pools	Intertidal areas	Sublittoral
** **Form	FSA, CM	T					CM	ASP, FF,
** **Distribution		Atlantic, Mediterranean, Indo-Pacific	Tropical to warm temperate	Tropical to warm temperate	Atlantic, Mediterranean, Indo-Pacific	Tahiti (type locality), Hawaiian Is., Southern Japan, Europe	Tropical to warm temperate	Tropical to warm temperate
**Reference**	Present study	[34]	[32]	[31]	[34]	[33]	[32]	[31]

ACD, Apical cell division; ASP, Attached spongy pompons; CM, Loose lying cushion-like mats; FF, Floating fluffs; FSA, Floating spherical aggregations; ICD, Intercalary cell division; T, Turf

To our knowledge, this is the first record of *C*. *socialis* having a floating spherical aggregation form, as previous reports have only described delicate felts a few mm high growing on rocks and crustose corallines, high mats and pompons growing on intertidal rocks, and fluffs floating in salt marsh and mangrove lagoons [31, 32].

*Aegagropila linnaei* can occur in several different forms, such as a mat-like attached form, a floating unattached mat form, free-living filaments, and spherical aggregations depending on environmental conditions [4–6, 35–37]. In *A*. *linnaei* the ball shape is the result of both mechanical factors, such as wave-induced rolling motion, and morphological features leading to entanglement of the filaments [1, 6, 35, 37]. In this study, different *C*. *socialis* growth forms were also observed in different water motion environments, e.g., floating spherical aggregations and mat-like forms with semi-spherical aggregations were observed on the shore on the downwind side where there was water movement. Externally, members of the study species were entangled in each other; consequently, branches growing in different directions were found within a clump. Similar to *A*. *linnaei*, our results suggest that water movement and morphological characteristics promote the formation of spherical aggregations in *C*. *socialis* (Fig 3). Detailed surveys of the physical underwater environment and laboratory culture experiments will be required in the future to clarify the mechanism underlying spherical aggregation formation.

**Fig 9:**
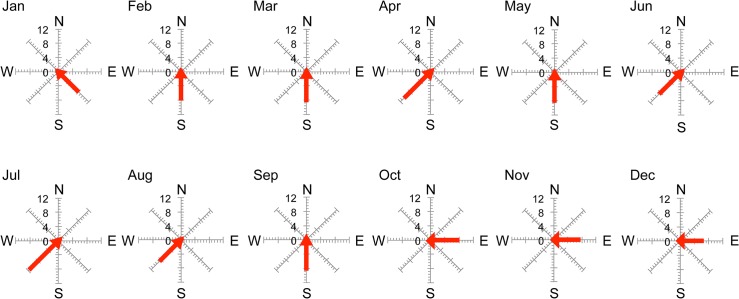
Monthly maximum velocity (m s^−1^) and wind direction in Central Thailand. Graphs were drawn from raw data provided by the Thai Meteorological Department [38].

Wind is one of the main factors causing movement in stagnant waters that have no regular in- or outflow. We suspect that wind direction does not influence water movement (which induces spherical aggregation) at the study site because the wind direction was same in July, when the spherical aggregations occurred, as it was in August when they disappeared (Fig 9). However, differences in wind velocity are more likely to influence water movement and spherical aggregation formation because wind velocity in July (11 m s^−1^) was different to that in August (8 m s^−1^). Additionally, wind velocity was fastest in July that year (Fig 9) [38]. Thus, faster wind velocity induces stronger water movement, and stronger water movement may promote spherical aggregation formation in this seaweed. However, according to the owner of this reservoir, mass occurrence of spherical aggregations does not occur every year and is a very rare phenomenon. Because their occurrence is sudden, short-term, and rare, it is unlikely that spherical aggregations only occur because of water movement promoted by wind; other physical conditions are most likely involved.

Although *Cladophora* is the largest green algae genus [13, 14], information on their internal morphology and ultrastructure is limited [22, 23, 39–44]. *Cladophora* pyrenoid ultrastructure is divided into four types; bilenticular, zonal, simple polypyramidal, and complex polypyramidal [23]. The bilenticular type, which is composed of a matrix traversed by a single thylakoid band, has been reported in *C*. *socialis* [31]. Additionally, a zonal type in which the pyrenoid matrix is divided by parallel thylakoid bands was observed by TEM for the first time in this study. Both of these pyrenoids are common in *Cladophora* species.

Morphological classification does not necessarily always reflect molecular phylogenetic analysis [20, 45]. For example, *C*. *coelothrix* is not a monophyletic taxon, and it is distributed in a number of different clades in a phylogenetic tree [13, 20]. Thus, it is occasionally difficult for species level identification in *Cladophora* using part of a gene, such as the 18S rDNA and 28S rDNA nucleic sequences, because of limitations in classification sensitivity. The authors consider it important to develop whole genome identification methodologies in the future, such as the amplified fragment length polymorphism (AFLP) method, for more exact *Cladophora* species identification.

## Conclusion

A mass occurrence of spherical *Cladophora* aggregations appeared for a short time in a salt field reservoir during July 2013 in Central Thailand. Extensive populations of spherical aggregations floated, accumulated, and colonized the water surface offshore where there was water movement. Additionally, individual filaments within the aggregations were entangled in each other, consequently branches growing in different directions were found within a clump. We suggest that water movement and morphological characteristics promote the formation of the spherical aggregations. Based on its external morphology, ultrastructure, growth pattern, and the molecular sequencing analysis, we conclude that the species in question is *C*. *socialis*. This is the first record of *C*. *socialis* having a floating spherical aggregation form.

## Supporting Information

S1 Movie Floating *Cladophora socialis* spherical aggregations and mat-like forms with semi-spherical aggregations (underwater). These forms were observed offshore on the downwind side of a salt field reservoir.
(AVI)
